# Supplementary material for: The Therapeutic Effect of Phosphopeptide P140 Attenuates Inflammation Induced by Uric Acid Crystals in Gout Arthritis Mouse Model
Source: Cells. 2022 Nov 22;11(23):3709. doi: 10.3390/cells11233709 (PMC9740613; doi:10.3390/cells11233709)
Supplement: Supplementary file 1 [file cells-11-03709-s001.zip › cells-1982906-supplementary.pdf]

cells

Supplementary Material

The therapeutic effect of phosphopeptide P140 attenuates inflammation induced by uric acid crystals in gout arthritis mouse model.

Izabela Galvão[1], Dylan Mastrippolito[2], Mariana Aganetti[1], Victor Rocha[1], Laura Talamini[2], Cindy Verdot[2], Viviani Mendes[1], Vivian Louise Soares de Oliveira[3], Amanda Dias Braga[3], Vinicius Dantas[4], Ana Maria Caetano de Faria[4], Flávio A. Amaral[3], Philippe Georgel[5,6], Angélica T. Vieira[1,2*†] and Sylviane Muller[2,6,7*]

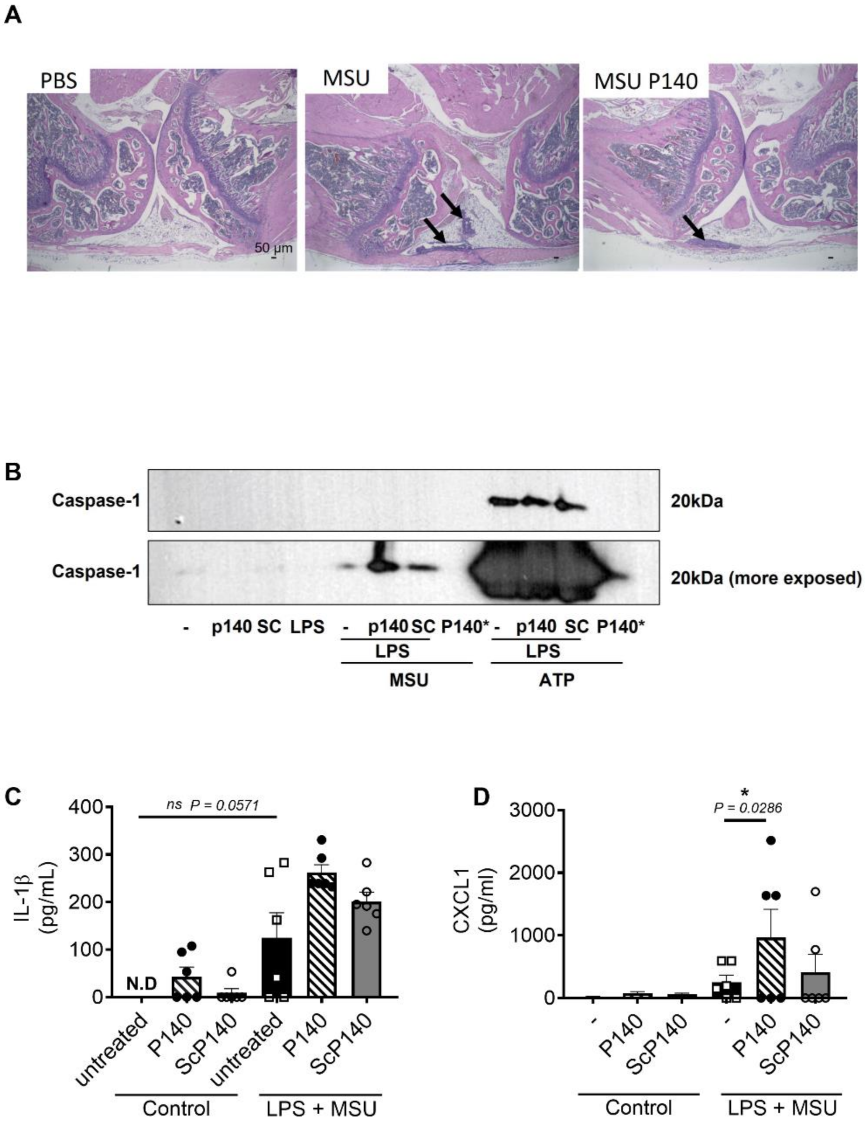

Figure S1. Effect of P140 peptide on inflammation. (**A**) Hematoxylin and eosin rapresentative pictures of knee cavity of PBS-, MSU-, MSU-P140- treated mice. Scale bar 50 µm. (**B,C,D**) BMDM were firstly treated with P140 or ScP140 (20µM) and secondly stimulated with LPS (1µg/mL) for 1h. Next MSU (300µg/ml) or ATP 5mM was added to the culture for 6h or 30 min respectively. Cell supernatants were then collected for further testing. (**B**) Western blot images of cleaved caspase-1 expression (two times of exposure, namely normal and long exposure, are shown). (**C,D**) Levels of IL-1β and

CXCL1 released measured by ELISA (expressed as pg/mL). These results are representative of 2 independent experiments. Data are mean ± SEM.

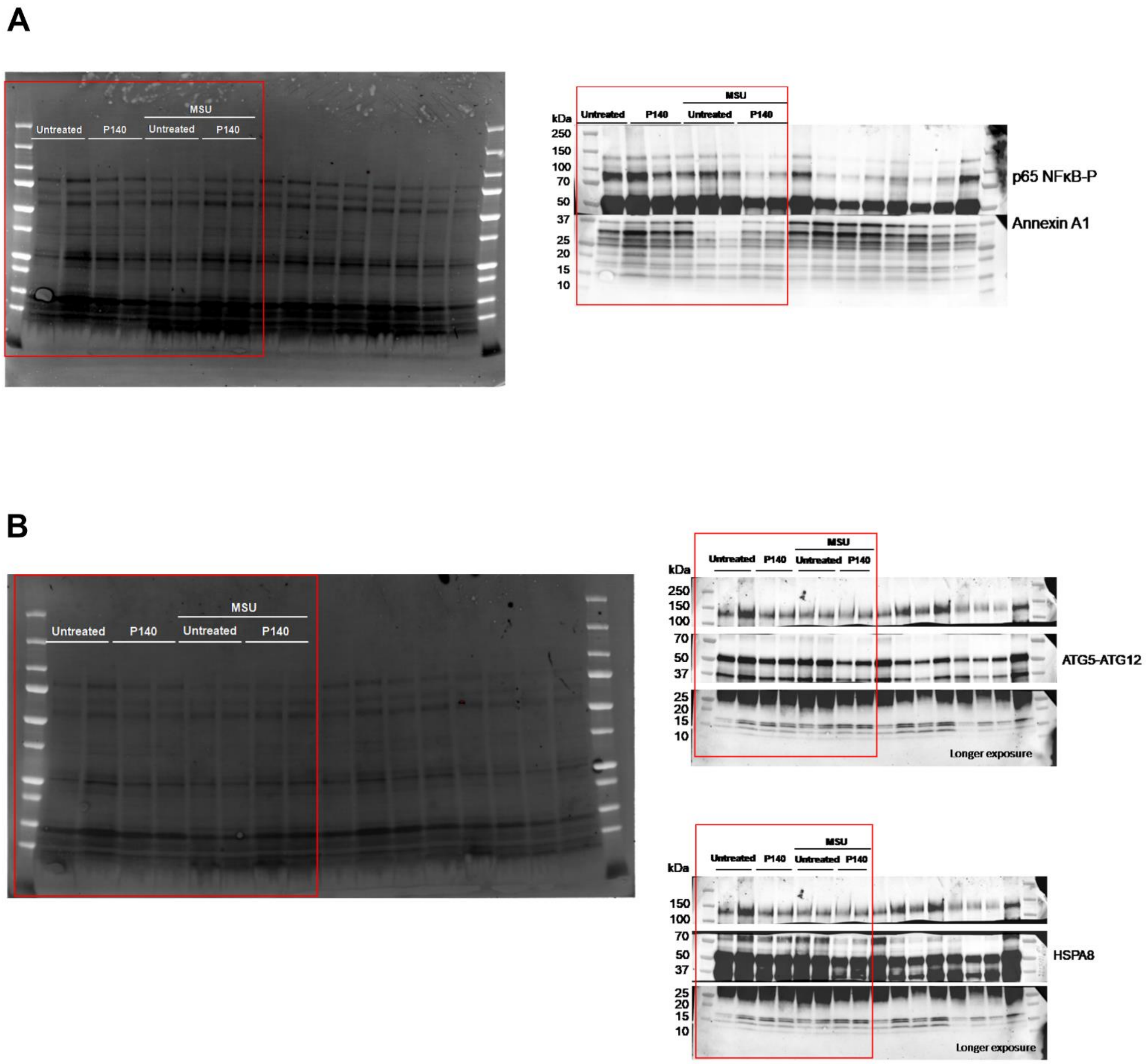

Figure S2: **Images of stain free blots used for protein quantification.** *In vitro* effect of P140 peptide on the expression of proteins of interest assessed by western blot. (**A**) Representative image of the stain-free used for total protein quantification and normalization (right panel). Membrane showing phosphorylated p65 NFκB and annexin A1 expression (left panel). (**B**) Representative image of the stain-free used for total protein quantification and normalization (right panel). Membrane revealing the expression of ATG5-ATG12 covalent complex and HSPA8 (left panel). To avoid quantification mistakes resulting from the fact that a loading control protein could represent a substrate for autophagy,

the expression levels of autophagy markers were done using stain-free technology (total protein lane content) using ImageJ or Image Lab software.
